# Antioxidative Activities of Both Oleic Acid and *Camellia tenuifolia* Seed Oil Are Regulated by the Transcription Factor DAF-16/FOXO in *Caenorhabditis elegans*

**DOI:** 10.1371/journal.pone.0157195

**Published:** 2016-06-08

**Authors:** Chia-Cheng Wei, Pei-Ling Yen, Shang-Tzen Chang, Pei-Ling Cheng, Yi-Chen Lo, Vivian Hsiu-Chuan Liao

**Affiliations:** 1 Department of Bioenvironmental Systems Engineering, National Taiwan University, Taipei, Taiwan; 2 Department of Forestry and Resource Conservation, National Taiwan University, Taipei, Taiwan; 3 Institute of Food Science and Technology, National Taiwan University, Taipei, Taiwan; National Cheng Kung University, TAIWAN

## Abstract

**Background:**

Tea seed oil is a high quality edible oil, yet lacking sufficient scientific evidences to support the nutritional and medical purposes. We identified major and minor components in *Camellia tenuifolia* seed oil and investigated the antioxidative activity and its underlying mechanisms in *Caenorhabditis elegans*.

**Principal Findings:**

The results showed that the major constitutes in *C*. *tenuifolia* seed oil were unsaturated fatty acids (~78.4%). Moreover, two minor compounds, β-amyrin and β-sitosterol, were identified and their antioxidative activity was examined. We found that oleic acid was the major constitute in *C*. *tenuifolia* seed oil and plays a key role in the antioxidative activity of *C*. *tenuifolia* seed oil in *C*. *elegan*s.

**Conclusions:**

This study found evidences that the transcription factor DAF-16/FOXO was involved in both oleic acid- and *C*. *tenuifolia* seed oil-mediated oxidative stress resistance in *C*. *elegans*. This study suggests the potential of *C*. *tenuifolia* seed oil as nutrient or functional foods.

## Introduction

Cooking oils, such as corn oil, palm oil, coconut oil, sunflower oil, olive oil, and sesame oil are daily consumed food and necessary for people. Among them, olive oil is highly consumed and developed as the nearly most widely applied edible oil around the world due to its high levels of monounsaturated fatty acids and polyphenol with beneficial effects [1, 2, 3]. In East Asia, tea seed oil has been used as high quality culinary oil for thousands of years, yet the scientific research for tea seed oil is limited. Tea seed oil (also known as tea oil or *Camellia* oil) is pressed from the seeds of *Camellia oleifera* and *Camellia tenuifolia*. In *C*. *oleifera* seed oil, the predominant fatty acids are the monounsaturated fatty acid (MUFA) oleic acid and the polyunsaturated fatty acid (PUFA) linoleic acid (LA) [4]. However, scientific data for *C*. *tenuifolia* seed oil is limited.

*Caenorhabditis elegans* (*C*. *elegans*) is a powerful genetic model organism to study biological processes such as cell division, development, oxidative stress, aging, and neuroscience [5, 6, 7]. *C*. *elegans* has become a popular model organism to investigate the beneficial effects from natural metabolites and products [8, 9]. In *C*. *elegans*, the sole forkhead transcription factor DAF-16 is the orthologue of FOXO family in mammals and in response to insulin/insulin-like growth factor 1 (IGF-1) signaling. DAF-16 activity is inhibited by phosphatidylinositol-3-OH kinase (PI3K)/protein kinase D (PDK)/Akt phosphorylation, which is regulated by insulin/IGF-I receptor DAF-2 [10, 11]. DAF-16 is a vital regulator and in response to environmental stimuli such as oxidative stress and heat shock [12, 13].

Oxidative stress up-regulates the transcription of antioxidant enzymes including superoxide dismutase (SOD), glutathione S-transferases (GST), and catalase by activating FOXOs [14]. Oxidative stress resulted from the accumulation of intracellular reactive oxygen species (ROS), causing the imbalance between the oxidant and antioxidant in organism, which in turn leading to DNA damage and various diseases [15]. In *C*. *elegans*, several oxidative stress resistance mechanisms have been investigated. For instance, SOD encoding genes (*sod-12345*) play an important role to reduce superoxide anion (O_2_^−^) to hydrogen peroxide (H_2_O_2_) [16]. In addition, catalase enzymes (CTL-123) and glutathione peroxidases (GPx) are responsible for detoxifying H_2_O_2_ to H_2_O or O_2_ [17]. Several studies have shown the protective effects from natural compounds against oxidative stress via DAF-16 regulation in *C*. *elegans* [18–20].

In the present study, we used *C*. *elegans* as an *in vivo* model organism to study the antioxidative properties of major and minor components in *C*. *tenuifolia* seed oil. Fatty acids contents in tea seed oil were analyzed by gas chromatograph (GC) and the antioxidant property of the corresponding level of unsaturated fatty acid oleic acid in *C*. *tenuifolia* seed oil was examined. In addition, compounds other than fatty acids were identified by liquid chromatography–mass spectrometry (LC-MS) and the antioxidant activity of the identified compounds was examined. Moreover, mechanistic aspect of antioxidant properties *in vivo* for oleic acid and seed oil from *C*. *tenuifolia* was dissected.

## Materials and Methods

### Chemicals

All chemicals unless otherwise stated were purchased from Sigma-Aldrich (St. Louis, MO, USA). Tea seeds were collected in November, 2013 from *C*. *tenuifolia* trees located in New Taipei City, Taiwan (with the permission of the land owner) and further cold-pressed to obtain tea seed oils. The species of *C*. *tenuifolia* trees were identified by the Taiwan Forestry Research Institute. Tea seed oil was dissolved in dimethyl sulfoxide (DMSO).

### Fatty acids analysis by gas chromatograph (GC)

Fatty acids of tea seed oil were analyzed by GC−flame ionization detection (GC-FID) using a gas chromatograph (GC 7890A, Agilent Technologies, Santa Clara, CA, USA) with a FID (Agilent Technologies) equipped with a 30 m × 0.25 mm × 0.25 μm DB-Wax column (J&W Scientific, Agilent Technologies). The temperatures of the injection port and the detector were 250 and 250°C, respectively. Samples were desorbed in the split mode (split ratio 20:1). The oven temperature program was held at 120°C for 1 min, then from 120 to 200°C at 10°C min^−1^, to 250°C at 4°C min^−1^, and finally held at 250°C for 3 min. Helium was used as the carrier gas at a flow rate of 1 ml min^−1^. The peak areas of the target compounds were used to quantify the absolute contents compared to that of calibration samples with known concentrations.

### Identification of compounds other than fatty acids by liquid chromatography–mass spectrometry (LC-MS)

The oil sample (0.1 g) was dissolved in 1 ml of *n*-hexane. A NH_2_ cartridge column was conditioned by passing of 6 ml of *n*-hexane. The dissolved oil solution was applied to the column, and then washed the sample column with 6 ml of *n*-hexane/ethyl acetate (95:5, v/v). Afterwards, the column was eluted sequentially with 5 ml of chloroform, acetone, and methanol. The 15 ml of eluted solvent was evaporated under a stream of N_2_ gas. Finally, the residue was dissolved with 5 ml of methanol, and filtered by 0.22 μm syringe filter. The final solution (10 μl) was injected into the LC system.

Chromatographic analysis was conducted with Acquity Ultra Performance LC system equipped with UV detector and MICROMASS Quattro Premier XE MS system (Waters, USA). A reversed-phase Waters BEH RP-18 column (2.1 mm × 100 mm i.d., particle size 1.7 μm) (Waters, USA) was used. Elution was performed at a flow rate of 0.2 ml/min. The mobile phase consisted of water (solvent A) and methanol (solvent B), both containing 0.1% formic acid. The column was balanced with the solvent ratio of 1:9 (water to methanol). The elution was further performed with gradient water-methanol from 1:9 (v/v) to 100% methanol, sequentially.

Mass spectra were scanned by atmospheric-pressure chemical ionization (APCI) in positive mode. The conditions were set up as follows: corona, cone and extractor voltages at 3kV, 25V and 4V, respectively. Source temperature was at 120°C. APCI probe temperature was at 450°C. Desolvation gas flow was at 700 l/hr, and cone gas flow was at 50 l/hr. The chemical compounds were identified, and the data were consistent with those described [21].

### *C*. *elegans* strains and culture conditions

*C*. *elegans* strains used in this study were Bristol N2 (wild-type); GR1307 *daf-16*(*mgDf50*); TJ356 zIs356(*daf-16*::GFP). All *C*. *elegans* strains and the *Escherichia coli* (*E*. *coli*) OP50 strain were obtained from the *Caenorhabditis* Genetics Center (CGC) (University of Minnesota, MN, USA), which is funded by the NIH National Center for Research Resources. *C*. *elegans* was maintained and assayed (unless otherwise stated) at 20°C on nematode growth medium (NGM) agar plates seeding with *E*. *coli* OP50 as a food source. Synchronization of worm cultures was achieved by using the hypochlorite protocol [22]. All chemicals in NGM plates and liquid are expressed as the final concentrations in this study.

### *C*. *elegans* oxidative stress resistance assays

Oxidative stress resistance assays were performed as previously described [9, 23] with slight modification. Synchronized L1 larvae of wild-type N2 or GR1307 *daf-16* mutant strain were incubated in liquid S-basal containing *E*. *coli* OP50 bacteria at 10^9^ cells/ml in the presence or absence of *C*. *tenuifolia* seed oil, oleic acid, β-amyrin, and β-sitosterol using DMSO (0.1%, v/v) as solvent control for 72 h at 20°C in dark. Subsequently, nematodes were exposed to 250 μM juglone (5-hydroxy-1, 4-naphthoquinone) [19] for 2.5 h [23, 24], and then scored for survival. The survival of worms was determined by touch-provoked movement as described [13]. Nematodes were scored as dead when they failed to respond to repeated touching with a platinum wire pick. At least 60 nematodes were examined per treatment and the test was performed at least 3 independent biological replicates.

### *C*. *elegans* intracellular reactive oxygen species (ROS) measurement

Wild-type N2 nematodes were raised from L1 larvae and pretreated with *C*. *tenuifolia* seed oil (0.01%, v/v) or oleic acid (0.066 mg/ml). After 72 h at 20°C incubation in dark, nematodes were washed with liquid S-basal medium three times and then transferred to 500 μL of phosphate buffered saline (PBS) containing 100 μM 2’,7’-dichlorodihydrofluoroscein diacetate (H_2_DCFDA) (Molecular Probes, Eugene, OR, USA) for 2.5 h at 20°C in dark. Before microscopic examination, worms were washed 3 times with PBS. At least 20 randomly selected worms from each set of experiments were mounted onto microscope slides coated with 2% agarose, anesthetized with 2% sodium azide, and capped with coverslips. Epifluorescence images were captured with an epifluorescence microscope (Leica, Wetzlar, Germany) using a suited filter set (excitation, 480 ± 20 nm; emission, 510 ± 20 nm) with a cooled charge-coupled device (CCD) camera. Total fluorescence for each whole worm was quantified by Image-Pro Plus software (Media Cybernetics, Bethesda, MD, USA). The test was performed at least 3 independent biological replicates.

### Subcellular DAF-16 localization

Synchronized L1 larvae of the transgenic strain TJ356 (expressing a DAF-16::GFP fusion protein) [25] were incubated in liquid S-basal containing *E*. *coli* OP50 bacteria at 10^9^ cells/ml and *C*. *tenuifolia* seed oil (0.01%, v/v), oleic acid (0.066 mg/ml), or DMSO (0.1%, v/v) as solvent control for 72 h at 20°C in dark, then the worms were challenged by 50 μM juglone treatment for 5 min. Worms were then placed on microscope slides coated with 2% agarose, anesthetized with 2% sodium azide, capped with coverslips, and then the subcellular DAF-16 distribution was analyzed by fluorescence microscopy on an epifluorescence microscope (Leica). The expression patterns of TJ356 worms were classified into three categories (cytosolic, intermediate, and nuclear) with respect to the major localization of the DAF-16::GFP fusion protein as previously described [25]. Subcellular DAF-16 localization was examined in approximately 30 animals per condition. The test was performed at least 3 independent biological replicates.

### Statistical analysis

Statistical analysis was performed using SPSS Statistics 22.0 Software (SPSS, Inc., Chicago, IL, 2013). The results are presented as the mean ± standard errors of mean (SEM). Comparisons and *P* value calculations were performed between treated and untreated animals using one-way ANOVA test. Differences were considered significant at *P* < 0.05 (see figures).

## Results and Discussion

### *C*. *tenuifolia* seed oil enhanced juglone-induced oxidative stress resistance in *C*. *elegans*

**Fig 1:**
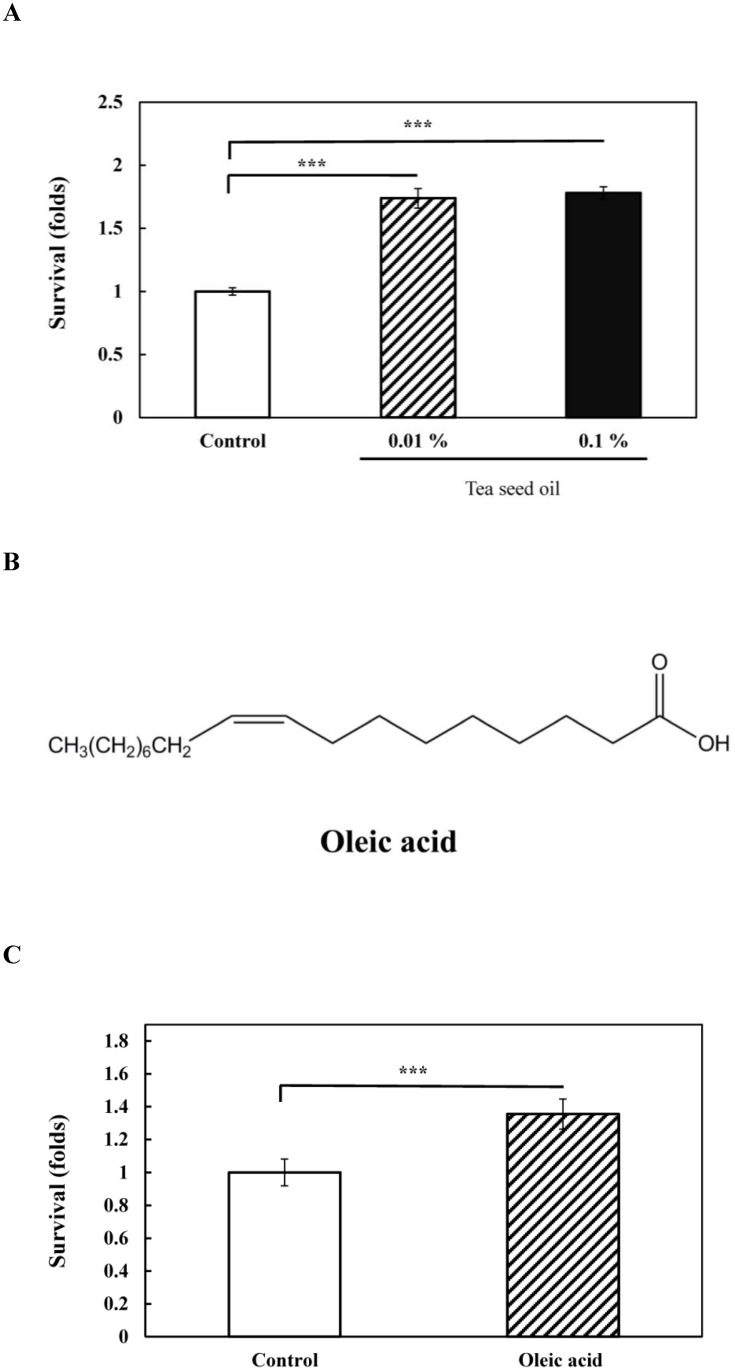
Antioxidative effects of *C*. *tenuifolia* seed oil and oleic acid on wild-type *C*. *elegans* N2 under oxidative stress. (A) Tea seed oil treated (0.01 and 0.1%) and untreated DMSO solvent control nematodes were exposed to 250 μM juglone for 2.5 h, and then scored for the survival. (B) The structure of monounsaturated fatty acid, oleic acid. (C) Oleic acid treated (0.066 mg/ml) and untreated DMSO solvent control nematodes were exposed to 250 μM juglone for 2.5 h, and then scored for the survival. At least 3 independent biological replicates were performed, and at least 60 worms were scored in each experiment. Data are normalized to the untreated DMSO solvent control. Results are presented as the mean ± standard error of mean (SEM). Differences compared to the untreated DMSO solvent control were considered significant at *p* < 0.001 (***) by one-way ANOVA test.

Common edible oils such as olive and sesame oils have been reported to have antioxidative activities [26, 27]. To investigate whether *C*. *tenuifolia* seed oil has antioxidant property *in vivo*, we conducted the oxidative stress assays in *C*. *elegans*. In order to reflect the true diet behavior of edible oil, we initially used tea seed oil which was pressed from the seed of *C*. *tenuifolia* for the assays. Wild-type N2 *C*. *elegans* was pretreated with 0.01 and 0.1% of *C*. *tenuifolia* seed oil followed by exposures to oxidative stress generator, juglone. The results showed that the pretreatments with *C*. *tenuifolia* seed oil (0.01 and 0.1%) significantly increased the survival of *C*. *elegans* after exposing to juglone (for 0.01%, 1.74 folds higher than the DMSO solvent control, *p* < 0.001; for 0.1%, 1.78 folds higher than the DMSO solvent control, *p* < 0.001) (Fig 1A). The result demonstrated that *C*. *tenuifolia* seed oil exerted significant antioxidant activities *in vivo*, and both 0.01 and 0.1% *C*. *tenuifolia* seed oil showed similar performance. We then conducted further experiments with the concentration of 0.01% of *C*. *tenuifolia* seed oil.

### Fatty acids in *C*. *tenuifolia* seed oil

We further investigated which constitutes in *C*. *tenuifolia* seed oil might play essential roles in the aforesaid antioxidative stress activity in *C*. *elegans*. It has been reported that major constituents of edible oils are fatty acids, which determine the quality and health characteristic of oils [28]. Therefore, we analyzed the fatty acids composition in tea seed oil from *C*. *tenuifolia* by GC-FID. In addition, several studies have reported that the major types of fatty acids in *Camellia* oil are oleic acid (OA) (C18:1), linoleic acid (LA) (C18:2), stearic acid (SA) (C18:0), and palmitic (PA) (C16:0) [4, 29, 30]. We therefore selected those aforesaid fatty acids for the analysis in *C*. *tenuifolia* seed oil.

The results showed that *C*. *tenuifolia* seed oil contained 864.5 ± 19.4 mg/g (86.5 ± 1.9%) total fatty acids, in which the unsaturated fatty acids were 78.4 ± 1.4% (Table 1). The monounsaturated fatty acid, oleic acid (C18:1) (Fig 1B), was the major type of fatty acid with 662.3 ± 12.4 mg/g (66.2 ± 1.2%) in *C*. *tenuifolia* seed oil (Table 1). The high contents of unsaturated fatty acids with oleic acid as the major type of fatty acid in *C*. *tenuifolia* seed oil were in agreed with the fatty acid compositions in *C*. *oleifera* seed oil [29–31]. Yet other bioactive components in *C*. *tenuifolia* seed oil such as *Camellia* saponin, tea polyphenol, squalene remain further identified and investigated for their biological roles.

**Table 1:** Fatty acids contents in *C*. *tenuifolia* seed oil in this study.

Fatty acids	mg/g	% (w/w)
**Total fatty acids**	**864.5 ± 19.4**	**86.5 ± 1.9**
**Saturated**	**80.3 ± 5.3**	**8.0 ± 0.3**
Palmitic acid (C16:0)	61.6 ± 1.9	6.2 ± 0.2
Stearic acid (C18:0)	18.7 ± 3.5	1.9 ± 0.4
**Unsaturated**	**784.2 ± 14.1**	**78.4 ± 1.4**
Oleic acid (C18:1)	662.3 ± 12.4	66.2 ± 1.2
Linoleic acid (C18:2)	122.0 ± 1.7	12.2 ± 0.2
α-Linolenic acid (C18:3)	ND ^a^	ND ^a^

Results are presented as the mean ± standard error of mean (SEM).

^a^ ND, not detectable.

### Oleic acid enhanced juglone-induced oxidative stress resistance in *C*. *elegans*

Oleic acid has attracted great attention, as the "Mediterranean diet" was characterized by a high olive oil (rich in OA) consumption [32]. Oleic acid has been linked to have beneficial effects on cardiovascular disease and serum lipids and protective effects against cancer [33, 34], yet the role of oleic acid on oxidative stress resistance was less described.

Since oleic acid is the major type of unsaturated fatty acid in *C*. *tenuifolia* seed oil, we further investigated whether oleic acid plays an important role in oxidative stress resistance in *C*. *tenuifolia* seed oil. Wild-type N2 *C*. *elegans* was pretreated with oleic acid (0.066 mg/ml, the corresponding level in 0.01% *C*. *tenuifolia* seed oil) followed by exposure to 250 μM juglone for 2.5 h. Fig 1C showed that the pretreatments with oleic acid significantly increased the survival of *C*. *elegans* when exposed to juglone (around 1.36 folds higher than the DMSO solvent control group, *p* < 0.001). This suggests that oleic acid played an important role in protecting *C*. *elegans* from juglone-induced oxidative stress, whereas remaining unidentified components such as phenolic compounds may also contribute to the increased oxidative stress resistance in the oxidative stress assays.

### The effects of oleic acid and *C*. *tenuifolia* seed oil on intracellular ROS level in *C*. *elegans*

**Fig 2:**
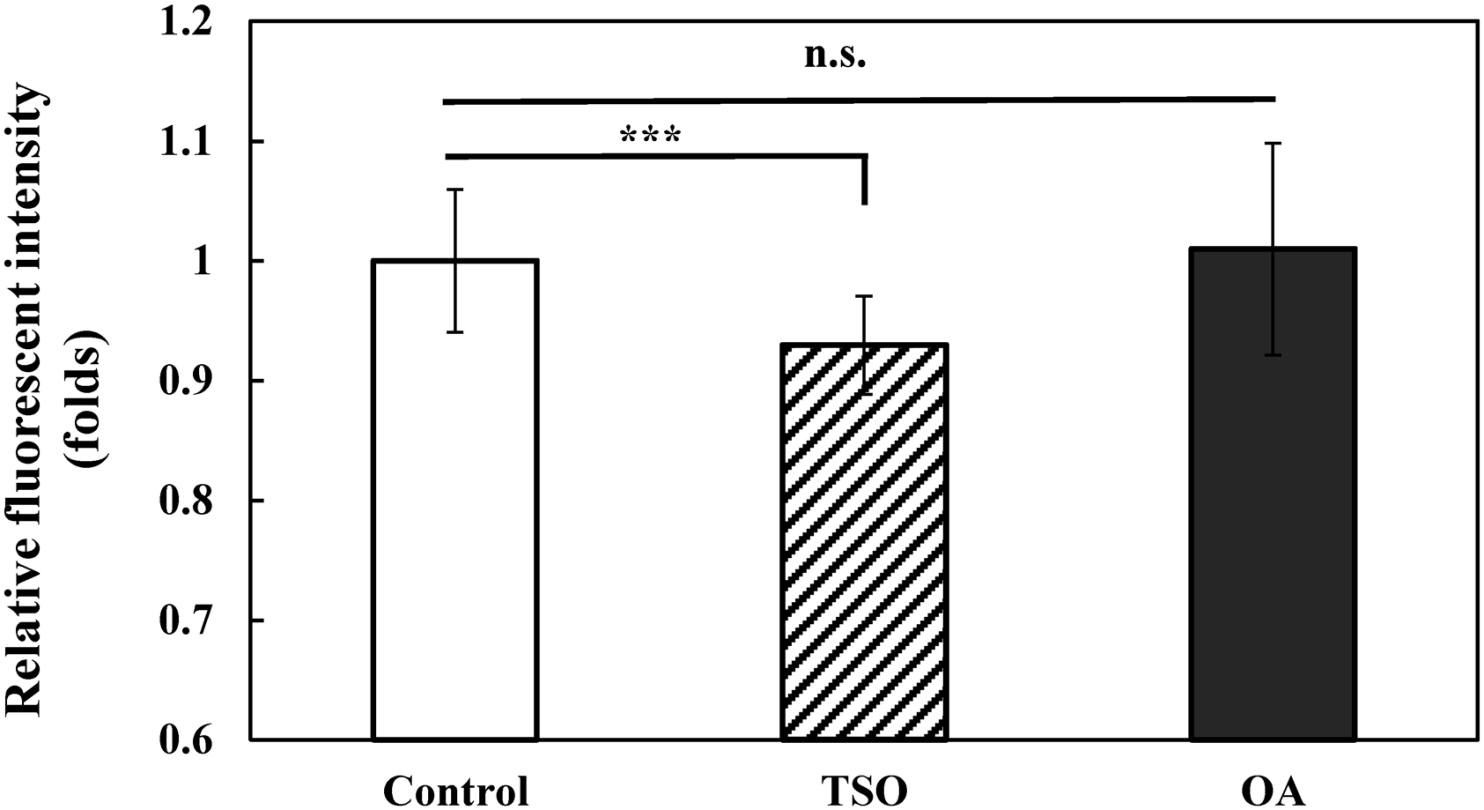
Effects of *C*. *tenuifolia* seed oil and oleic acid on intracellular reactive oxygen species (ROS) production in wild-type *C*. *elegans* N2. ROS level after pretreated with tea seed oil (TSO) (0.01%), oleic acid (OA) (0.066 mg/ml). At least 20 randomly selected worms from each set of experiments were directly measured for the total GFP fluorescence for each whole worm. At least 3 independent biological replicates were performed. Data are normalized to the untreated DMSO solvent control. Results are presented as the mean ± standard error of mean (SEM). Differences compared to the untreated DMSO solvent control were considered significant at *p* < 0.001 (***) by one-way ANOVA test. n.s., not significant.

To investigate whether *C*. *tenuifolia* seed oil or oleic acid suppressed oxidative stress in *C*. *elegans* was due to ROS scavenging ability, intracellular ROS production in *C*. *elegans* after *C*. *tenuifolia* seed oil (0.01%) or oleic acid (0.066 mg/ml) pretreatment was measured. We applied non-fluorescent H_2_DCFDA cell-permeable dye to detect ROS level by which interaction with intracellular H_2_O_2_ to form detectable fluorescent 2’7’-dichlorofluorescein (DCF) [35]. The results showed that *C*. *tenuifolia* seed oil (0.01%) significantly reduced the level of ROS compared to that untreated DMSO solvent control (*p* < 0.001) (Fig 2). This suggests that pretreatment of *C*. *tenuifolia* seed oil reduced oxidative stress-induced toxicity (Fig 1A) by decreasing the intracellular ROS production in *C*. *elegans* (Fig 2). In contrast, the level of ROS between oleic acid (0.066 mg/ml) and untreated DMSO solvent control was not significantly different (Fig 2). This suggests that the observed effect of reduced ROS level by *C*. *tenuifolia* seed oil might be contributed from other bioactive constituents, not mainly from the major constituent oleic acid. In addition, other ROS such as NO (nitric oxide) and O_2_^−^ (oxide anion) which could not be detected by the H_2_DCFDA dye, might involve in oleic acid suppressed oxidative stress.

### DAF-16 was involved in oleic acid and *C*. *tenuifolia* seed oil mediated oxidative stress resistance in *C*. *elegans*

We further investigated the possible underlying mechanisms by *C*. *tenuifolia* seed oil oleic acid and *C*. *tenuifolia* seed oil mediated oxidative stress resistance. In *C*. *elegans*, the sole forkhead transcription factor, DAF-16, is a vital regulator and in response to environmental stimuli such as oxidative stress and heat shock [12, 13]. In order to determine whether DAF-16 is involved in *C*. *tenuifolia* seed oil and oleic acid mediated stress resistance in *C*. *elegans*, we investigated its effects on juglone-induced oxidative stress by the *daf-16* deletion mutant strain GR1307. The presumption is that if DAF-16 is required for oxidative stress resistance induced by tea seed oil or oleic acid, the antioxidative effect would not be observed in *daf-16* null mutant that was treated with tea seed oil or oleic acid.

**Fig 3:**
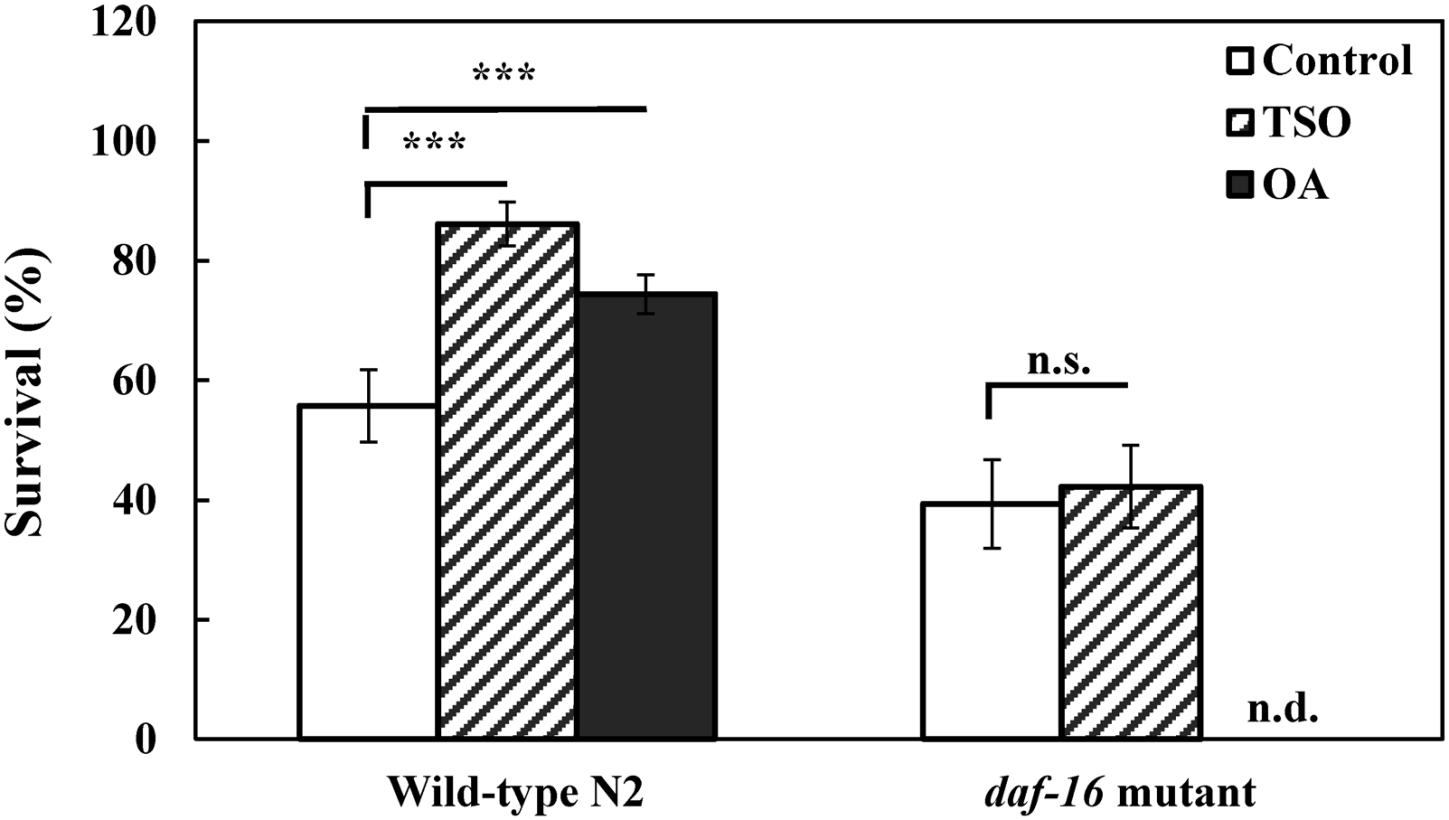
Effects of *C*. *tenuifolia* seed oil and oleic acid on *daf-16* mutant strain GR1307 under oxidative stress. Wild-type and *daf-16* mutant nematodes were raised and treated with *C*. *tenuifolia* seed oil (TSO) (0.01%) and oleic acid (OA) (0.066 mg/ml).as described above and subjected to oxidative stress assay as described above and scored for survival of worms. At least 3 independent biological replicates were performed, and at least 60 worms were scored in each experiment. Results are presented as the mean ± standard error of mean (SEM). Differences compared to the untreated DMSO solvent control were considered significant at *p* < 0.001 (***) by one-way ANOVA test. n.s., not significant.

The *daf-16* mutant worms were raised from L1 larvae for 72 h at 20°C and followed by juglone-induced oxidative stress as the stress resistance assays for wild-type worms. The results showed that the survival of *daf-16* mutant worms which were treated with *C*. *tenuifolia* seed oil (0.01%) was not significantly different from the survival of *daf-16* mutant worms which were not treated with *C*. *tenuifolia* seed oil (Fig 3). This suggests that the ability to increase oxidative stress resistance by tea seed oil in *daf-16* mutant worms was not obvious as the wild-type worms (Fig 3). Similar to several studies on natural antioxidants in *C*. *elegans* [19, 36], our result suggests that the forkhead transcription factor DAF-16 was involved in regulation of oxidative stress resistance enhanced by *C*. *tenuifolia* seed oil.

We also treated *daf-16* mutant worms with oleic acid (0.066 mg/ml), but the toxic effects such as delayed *C*. *elegans* growth and decreased population of worms were observed under purely oleic acid exposure. This is possible as several studies have shown that DAF-16 regulates fatty acid metabolism in *C*. *elegans* [14, 37, 38]. Therefore, the observed adverse effect in *daf-16* mutant is indispensable for additional treatment of oleic acid regulation in *C*. *elegans*.

### *C*. *tenuifolia* seed oil and oleic acid influence subcellular DAF-16 localization

**Fig 4:**
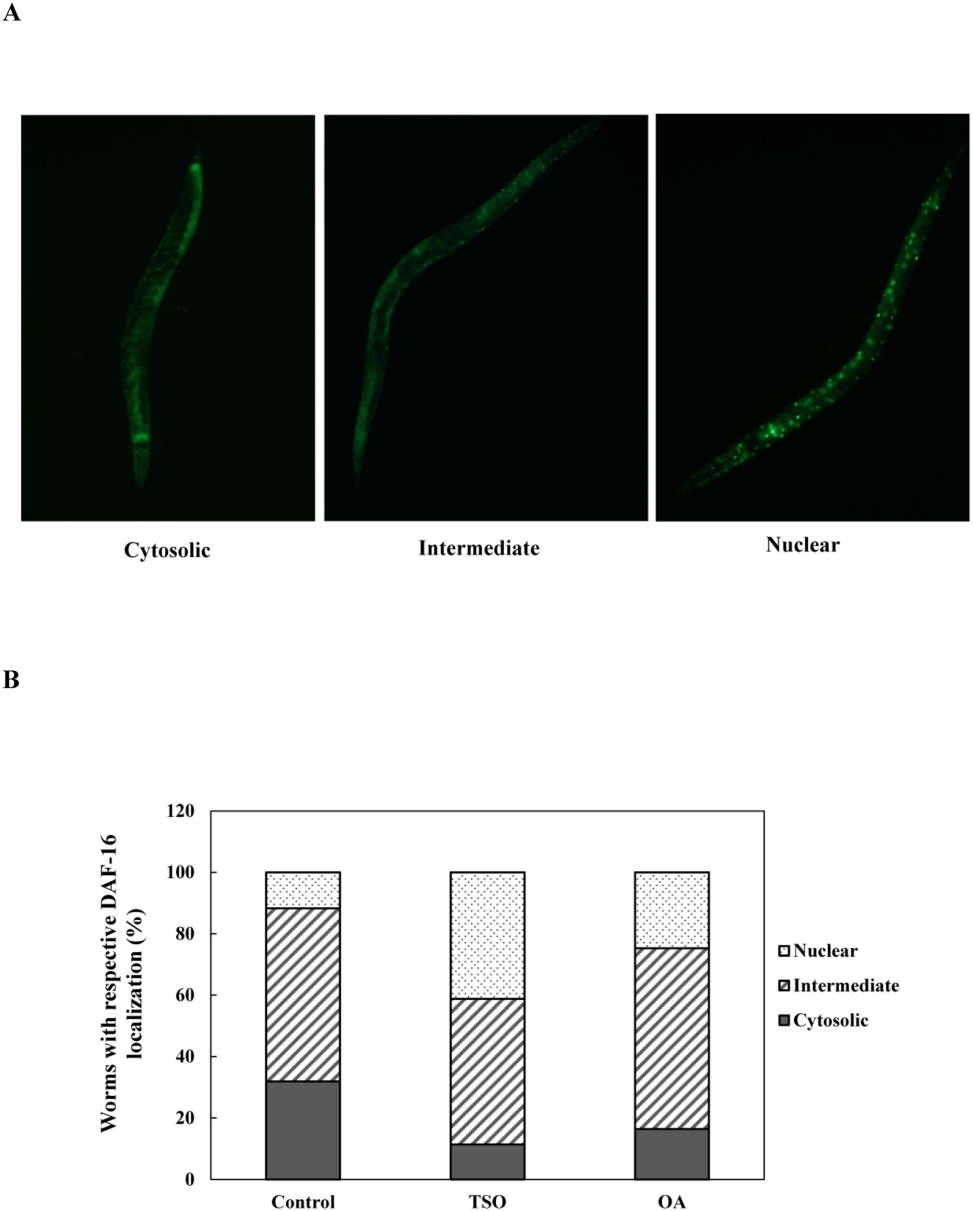
Influences of *C*. *tenuifolia* seed oil and oleic acid on subcellular DAF-16 localization. Strain TJ356 was raised and treated with *C*. *tenuifolia* seed oil (TSO) (0.01%) and oleic acid (OA) (0.066 mg/ml).as described above and challenged by 50 μM juglone for 5 min at 20°C and the fluorescence intensity of worms was then scored. (A) The localizations of DAF-16::GFP could be classified into three categories: cytosolic (left), intermediate (middle), and nucleus (right). The presented phenotypes are pictured from the DMSO solvent control group. (B) Subcellular DAF-16 localization was scored in approximately 30 animals per condition and at least 3 independent biological replicates were performed. The phenotype results were presented in ratio to the whole population at each treatment condition.

It has been reported that DAF-16 nuclear localization is an essential key point for its ability to activate oxidative stress resistance in *C*. *elegans* [39, 40]. We postulated that the oxidative stress resistance ability of *C*. *elegans* by *C*. *tenuifolia* seed oil or oleic acid was due to the increased nuclei subcellular localization of DAF-16. Hence, we examined the translocation of DAF-16 by using the transgenic strain TJ356 (DAF-16::GFP). The localizations of DAF-16::GFP could be classified into 3 categories: cytosolic, intermediate, and nucleus and the representative phenotypes were presented in Fig 4A. As shown in Fig 4B, both *C*. *tenuifolia* seed oil (0.01%) and oleic acid (0.066 mg/ml) treated transgenic strain TJ356 showed increased fractions of nuclear localization of DAF-16 phenotype compared to the untreated DMSO solvent controls. The percentage of nuclear localization phenotype was increased from 12% to 41 and 25% with *C*. *tenuifolia* seed oil and oleic acid treatment, respectively (Fig 4B). The results of this assay indicates that *C*. *tenuifolia* seed oil and oleic acid enhanced translocation of DAF-16 from the cytoplasm to nuclei which was able to trigger the transcription of the downstream genes.

Although DAF-16 has been reported as key regulator for some natural products mediated stress resistance in *C*. *elegans* [19, 36], some studies in polyphenol have shown controversial results. For example, it has been suggested that the increase of stress resistance and lifespan of *C*. *elegans* by polyphenol quercetin was mediated by DAF-16 based on the results of subcellular distribution of DAF-16 [41]. In contrast, it has been reported that DAF-16 is not obligatorily required for polyphenol quercetin mediated stress resistance and lifespan extension in *C*. *elegans* due to quercetin also increased thermal and oxidative stress resistance and lifespan in *daf-16*(mgDf50) mutant worms [42]. In this study, we provided evidences from both *daf-16* null mutant strain and DAF-16 localization supporting that *C*. *tenuifolia* seed oil and oleic acid enhance oxidative stress resistance which was regulated by the forkhead transcription factor DAF-16 in *C*. *elegans*.

Edible oils with high level of oleic acid and low level of linoleic acid are considered with higher oxidative stability and can be used as a natural antioxidant in food stability [4, 30, 43]. Several studies reported that olive oil has multiple beneficial effects, including reduced cardiovascular disease, cognitive impairment, antioxidant activity, antimicrobial activity, and anti-inflammatory because of the high levels of monounsaturated fatty acids and polyphenol in olive oil [1, 2, 44–46]. Our study showed that the oleic acid content in *C*. *tenuifolia* seed oil (~66%) is comparable to that of olive oil (58.5–83.2%) [1, 47]. Although besides fatty acids, other compounds in *C*. *tenuifolia* seed oil remain to be further identified and characterized, our present study demonstrated that *C*. *tenuifolia* seed oil has the potential in nutritional supplement and medical application for oxidative stress related diseases.

### Identification and the antioxidant activity of compounds other than fatty acids in *C*. *tenuifolia* seed oil

**Fig 5:**
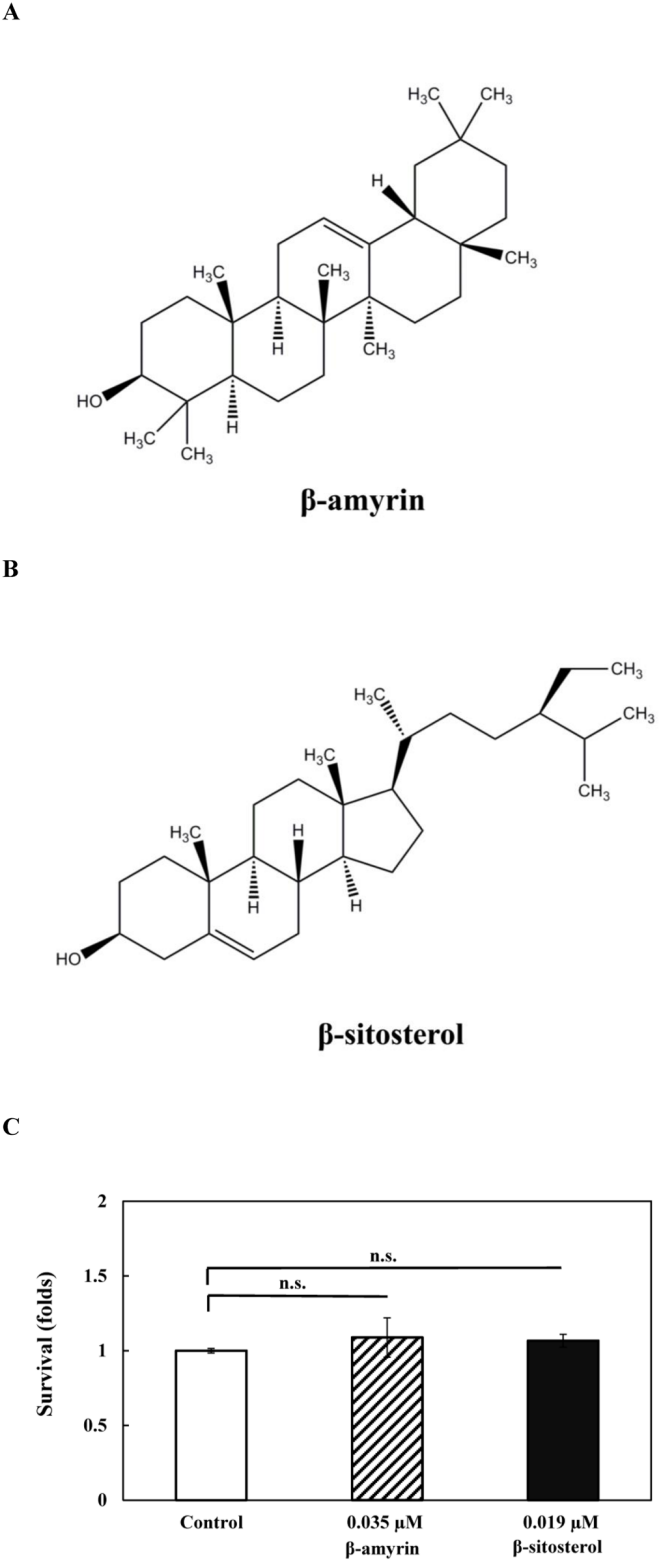
Antioxidative activities of β-amyrin and β-sitosterol in *C*. *tenuifolia* seed oil on wild-type *C*. *elegans* N2. (A) The structure of β-amyrin. (B) The structure of β-sitosterol. (C) β-amyrin- (0.035 μM, the corresponding level in 0.01% *C*. *tenuifolia* seed oil) and β-sitosterol-treated (0.019 μM, the corresponding level in 0.01% *C*. *tenuifolia* seed oil), and untreated DMSO solvent control nematodes were exposed to 250 μM juglone for 2.5 h, and then scored for the survival of worms. At least 3 independent biological replicates were performed, and at least 60 worms were scored in each experiment. Data are normalized to the untreated DMSO solvent control. Results are presented as the mean ± standard error of mean (SEM). Differences compared to the untreated DMSO solvent control were considered significant by one-way ANOVA test. n.s., not significant.

In addition to fatty acids, several bioactive compounds have been reported in *Camellia* tea seed oil, such as triterpenes (e.g., β-amyrin) and phytosterols (e.g., stigmasterol, β-sitosterol) [31, 48]. To investigate whether these bioactive compounds including β-amyrin, stigmasterol, and β-sitosterol are present in *C*. *tenuifolia* seed oil, LC-MS was used for the analysis. The results showed that β-amyrin (Fig 5A) and β-sitosterol (Fig 5B) were detected whereas stigmasterol was not detected in *C*. *tenuifolia* seed oil in this present study (Table 2). The concentrations of β-amyrin and β-sitosterol were about 0.1491 and 0.0789 mg/g, respectively in *C*. *tenuifolia* seed oil (Table 2). The relative contents of β-amyrin and β-sitosterol in *C*. *tenuifolia* seed oil were lower than that in *C*. *sinensis* seed oil, but β-amyrin was higher than that in olive oil [48].

**Table 2:** Compounds other than fatty acids in *C*. *tenuifolia* seed oil in this study.

Compounds	mg/g	% (w/w)
β-amyrin	0.1491 ± 0.0202	0.0149 ± 0.002
β-sitosterol	0.0789 ± 0.0223	0.0079 ± 0.0022
stigmasterol	ND ^a^	ND ^a^

Results are presented as the mean ± standard error of mean (SEM).

^a^ ND, not detectable.

In order to study the antioxidant activity of β-amyrin and β-sitosterol in *C*. *elegans*, we further conducted the oxidative stress resistance assays. Wild-type N2 *C*. *elegans* was pretreated with β-amyrin (0.035 μM, the corresponding level in 0.01% *C*. *tenuifolia* seed oil) and β-sitosterol (0.019 μM, the corresponding level in 0.01% *C*. *tenuifolia* seed oil) followed by exposure to 250 μM juglone for 2.5 h. Fig 5C showed that the pretreatments with β-amyrin and β-sitosterol at the examined concentrations did not significantly increase the survival of *C*. *elegans* when exposed to juglone-induced oxidative stress. This suggests that the individual minor compound (β-amyrin or β-sitosterol) at the corresponding concentration in 0.01% *C*. *tenuifolia* seed oil did not attribute to the antioxidant activity in *C*. *elegans* (Fig 5C).

Several studies reported β-amyrin and β-sitosterol with good antioxidant activities [49–53], but their effective concentrations were much higher than that in 0.01% *C*. *tenuifolia* seed oil. Therefore, it is unlikely that the minor compounds β-amyrin and β-sitosterol are individually responsible for the antioxidant activity provided by *C*. *tenuifolia* seed oil, unless the concentrations need to be much higher. This suggests that the major antioxidative stress contribution in *C*. *tenuifolia* seed oil was from oleic acid and possibly from other unidentified compounds which require further study.

## Conclusions

This study demonstrated that oleic acid and *C*. *tenuifolia* seed oil exerted excellent antioxidative stress activity *in vivo*. The unsaturated oleic acid was found as the major constitute in *C*. *tenuifolia* seed oil which is comparable to that of olive oil. Furthermore, the antioxidant activity of *C*. *tenuifolia* seed oil and oleic acid in *C*. *elegans* was regulated by the forkhead transcription factor DAF-16/FOXO. The novel aspect of this study is that to the best of our knowledge, this is the first study to report antioxidant activity of *C*. *tenuifolia* seed oil and its corresponding genetic mechanisms in intact model organism. The significance of this study is that the findings of this study provide scientific evidences and fundamental knowledge of pharmacological application of *C*. *tenuifolia* seed oil diet and suggest the potential of *C*. *tenuifolia* seed oil as nutrient or functional foods.
